# Knowledge mobilisation for chronic disease prevention: the case of the Australian Prevention Partnership Centre

**DOI:** 10.1186/s12961-018-0379-9

**Published:** 2018-11-16

**Authors:** Sonia Wutzke, Samantha Rowbotham, Abby Haynes, Penelope Hawe, Paul Kelly, Sally Redman, Seanna Davidson, Jackie Stephenson, Marge Overs, Andrew Wilson

**Affiliations:** 1 The Australian Prevention Partnership Centre, The Sax Institute, Ultimo, NSW 2007 Australia; 2 Menzies Centre for Health Policy, Charles Perkins Centre, University of Sydney, Sydney, NSW 2006 Australia; 3 Population Health, ACT Government Health Directorate, GPO Box 825, Canberra City, ACT 2601 Australia; 4 The Sax Institute, Ultimo, NSW 2007 Australia

**Keywords:** Knowledge mobilisation, knowledge to action, co-production, partnership research, chronic disease, prevention

## Abstract

**Background:**

Cross-sectoral, multidisciplinary partnership research is considered one of the most effective means of facilitating research-informed policy and practice, particularly for addressing complex problems such as chronic disease. Successful research partnerships tend to be underpinned by a range of features that enable knowledge mobilisation (KMb), seeking to connect academic researchers with decision-makers and practitioners to improve the nature, quality and use of research. This paper contributes to the growing discourse on partnership approaches by illustrating how knowledge mobilisation strategies are operationalised within the Australian Prevention Partnership Centre (the Centre), a national collaboration of academics, policy-makers and practitioners established to develop systems approaches for the prevention of lifestyle-related chronic diseases.

**Methods:**

We undertook interviews with key academics, policy, and practice partners and funding representatives at the mid-point of the Centre’s initial 5-year funding cycle. We aimed to explore how the Centre is functioning in practice, to develop a conceptual model of KMb within the Centre for use in further evaluation, and to identify ways of strengthening our approach to partnership research. Inductive and deductive thematic analysis was used to identify the key mechanisms underpinning the Centre’s KMb approach.

**Results:**

Six key mechanisms appeared to facilitate KMb within our Centre, namely Engagement, Partnerships, Co-production, Capacity and Skills, Knowledge Integration, and Adaptive Learning and Improvement. We developed a conceptual model that articulated these mechanisms in relation to the structures and processes that support them, as well as the Centre’s goals. Findings also informed adaptations designed to strengthen the Centre.

**Conclusions:**

Findings provide insights into the practical realities of operationalising KMb strategies within a research partnership. Overall, the centre is perceived to be progressing towards its KMb goals, but challenges include stakeholders from different settings understanding each other’s contexts and working together effectively, and ensuring knowledge generated across different projects within the Centre is integrated into a more comprehensive understanding of chronic disease prevention policy and practice. Our conceptual model is now informing ongoing developmental evaluation activities within the Centre, where it is being tested and refined.

## Background

### Research partnerships for knowledge mobilisation (KMb)

The value of research for improving health outcomes and optimising the use of health system resources is well recognised [1, 2]. However, traditional research production paradigms are often poor at providing rapid and relevant research that has the capacity for clinical, public health and policy impact [3]. For example, it is estimated that only 8–15% of research is applied in practice, with an estimated lag of up to 17 years between the development of research and its use [4–6]. Challenges to incorporating research in policy and practice are well documented and include decision-makers having insufficient time or capacity to read, interpret and apply evidence; the research not addressing relevant policy or practice questions or being insufficiently contextualised; lack of evidence availability when it is needed [7, 8]; cultural barriers between practitioners, policy-makers and researchers [9]; poor translation skills by researchers [6]; constraining organisational values, processes and incentives for KMb [10]; and wider challenges in the ever-changing political landscape [11, 12].

Cross-sectoral, multidisciplinary partnership research is one of the most effective means of facilitating research-informed policy and practice [4, 13, 14]. Research partnerships are especially helpful for addressing wicked problems where there are multiple interconnected causes, competing stakeholder interests and where the solutions are uncertain or unobtainable [14–16]. Underpinned by explicit KMb strategies, partnership research advocates a purposeful process of connecting academic researchers with decision-makers and practitioners with the goal of improving the relevance, quality and use of research in public policy and professional practice [17–20]. The inherent rationale of such approaches being that “*the best and most lasting influences of research come about not when information is linearly transferred to the practitioner, but when teams of practitioners and researchers co-create knowledge by working together*” [21].

### Mechanisms for KMb within research partnerships

While research partnerships have been found to increase the uptake of research in policy and practice [15, 22–24], there is no ‘gold standard’ for how such partnerships should be constituted or what strategies they should employ to maximise effectiveness. Nevertheless, increasing empirical evidence points to characteristics that strengthen the connectivity and positive outcomes of research partnerships. Table 1 provides a synthesis of 12 critical characteristics of effective partnerships identified from four different approaches to investigating the functionality of collaborations. These approaches include a case study of a Collaboration for Leadership in Applied Health Research and Care (CLAHRC) [25], a longitudinal realist evaluation of three CLAHRCs that had varied success [26], a systems-orientated review of collaborative KMb models in healthcare [27], and an experientially derived field guide to managing research collaborations from the ‘science of team science’ field [28].

**Table 1:** Collated overview of key characteristics of successful knowledge mobilisation partnerships

Characteristic	Description of characteristic	Heaton et al., 2016 [25]	Rycroft-Malone et al., 2016 [26]	Best & Holmes, 2010 [27]	Bennet et al., 2010 [28]
Structural and organisational features					
Effective governance and support	The collaboration develops agreed structures, roles and processes that facilitate connectivity and are supported by partner organisations	Collaboration partners have committed backing and receive strategic support from their respective organisations	Governance arrangements (structures and processes between people, places, ideology and activity) prompt opportunities for connectivity	The coordination infrastructure has agreed governance and task arrangements, including formalised rules, roles and structures	Projects meet regularly Roles and responsibilities are clear Partner organisations provide support ‘Prenuptial agreements’ document expectations
Influential leadership	Leaders foster connectivity They are credible, passionate, reflexive and empowering Local leaders forge connections and facilitate implementation	Project team leaders facilitate collaboration and knowledge mobilisation They are credible with solid connections, drive, enthusiasm and tenacity	Leadership is both formal and distributed/shared Leaders are reflexive Credible boundary spanners at different levels forge connections and facilitate implementation	Leadership style is collaborative and empowering Leaders model reflexivity and are responsive to emerging patterns of change	Leaders model collaborative skills They bring people together, listen, foster collegiality, build trust, empower and motivate members, share credit, and manage expectations
Supportive architecture	The collaboration has a clearly defined but flexible and responsive infrastructure Local project teams comprise key stakeholders	Project teams are formed around a small strategic core of end-users and researchers from partner organisations	The enterprise has a flexible structure and clear processes It is agile enough to support emergent ideas, relationships and processes	The enterprise has sufficient resources, capacity and role clarity to support good communication and management functions Partnering organisations provide time and resources	
Appropriate resourcing and rewards	The collaboration has (and builds) resources that foster connectivity, capacity and outcomes Assets are used to incentivise and reward members Partner organisations provide resources	Members develop assets to facilitate the enterprise, including particular knowledge and skills, routine data, platforms for shared learning, and publications	Resources (skills, funding, roles, opportunities, tools and artefacts) are positioned to catalyse engagement and outcomes Incentives appropriate to members’ contexts drive engagement		The collaboration uses its resources to ensure contributions are credited and rewarded Ownership of assets agreed in advance
Active conflict management	The collaboration tackles power imbalances, competition and conflicts via adaptable equity-focused processes and resource allocation		Competition and conflicting stakeholder agendas are addressed Resources are used to renegotiate and resolve the tensions caused by competition	Power disparities are recognised and addressed Negotiations use a process that is sensitive to power issues and sets fair expectations and ground rules	Leaders at all levels tackle conflicts Conflict management styles are adaptive Processes are in place to deal with disagreement
Mechanisms for knowledge mobilisation					
Leveraged pre-collaboration assets	Collaboration members make the most of existing relationships and other valuable assets	Members harness existing assets to facilitate the collaboration	The collaboration builds on pre-existing relationships and previous work and/or dialogue, thus achieving early ‘quick wins’		
Ownership and trust	The collaboration involves key stakeholders, including end-users, as active co-owners of the enterprise from beginning to end Trust is actively built	Research is driven by local end-users throughout the research life cycle Those developing research and intervention activities recognise that the key change agents are end-users	Research and implementation are owned by users resulting in co-production rather than knowledge transfer	Key stakeholders are represented in the main activities of the enterprise as active collaborators Trust is built over time	Trust is fostered through team-building activities, shared accountability, mentoring and leadership Members feel their contributions are valued
Shared vision and goals	Collaboration members negotiate and agree on the enterprise’s goals and outcomes	End-users and researchers have a common and coherent objective around which they coalesce	Stakeholders explore their understandings of outputs so motivation to collaborate is based on a shared view of goals and outcomes	Clear common aims are negotiated	Members understand the ‘big picture’ goals of the enterprise and know which goals they are working towards Expectations are discussed and agreed
Knowledge plurality and sharing	Different types of knowledge and experience are valued and used complementarily	Members and end-users meld different knowledge and expertise, valuing what each can contribute		Knowledge is viewed as plural, namely encompassing research and practice wisdom, tacit and explicit knowledge	Members respect each other’s input They share data and credit, and are willing to give and respond to feedback
Strategic communication	Communication is used to facilitate negotiations, share knowledge, build connectivity across boundaries and inspire change	New and more productive ways of working are identified and communicated	The benefits of collaboration are communicated and reinforced. Communications link projects across professional and epistemic boundaries	Ongoing communication tackles interdependencies, trade-offs and interests, builds mutual understanding and catalyses change	Leaders communicate clearly and decisively, share information and articulate the collaboration’s vision Members’ input is encouraged
Continuous learning and reflection	The collaboration continuously learns about itself (achievements, relationships, struggles, practices, opportunities) and acts on this knowledge productively	Members identify new and more productive ways of working, and apply these more widely Learning is actively shared with and adapted to kindred settings or populations	The collaboration has a reflective culture. It evaluates its progress and this information is acted on by leaders and other members, feeding into changes in ways of working	The collaboration continuously improves via feedback loops and reflective shared learning It recognises its knowledge as context-bound, multidirectional and emergent	Members are reflexive They strive to learn from the collaboration and improve their collaborative skills They recognise the collaboration and its goals will evolve
Capacity-building as a core activity	The collaboration provides resources and uses multiple strategies to build capacity	Professional development opportunities are created	Resources are used to maximise capacity building	The enterprise has and builds capacity	Mentors are cultivated Junior members’ career development is supported and collaboration skills are built

As illustrated in Table 1, successful research partnerships for KMb appear to be underpinned by a number of structural and organisational features, including effective governance and support for the roles, processes and activities to be undertaken; influential and credible leadership to foster connections across the partnership; conflict management processes; and resourcing to support and reward partnership activity. Across these research partnerships, a range of mechanisms also appear to be important in enabling KMb, including building ownership and trust between stakeholders, establishing a shared vision and goals, strategic communications to facilitate connections and share knowledge, continuous learning, reflection and adaptation, and capacity-building.

### The Australian Prevention Partnership Centre

The Australian Prevention Partnership Centre (hereafter the ‘Prevention Centre’ or ‘Centre’) [29] was established in June 2013 as an Australian National Health and Medical Research Council (NHMRC) Partnership Centre for Better Health [30]. Its goal is to identify systems, strategies and structures to inform better decisions about improving the prevention of lifestyle-related chronic disease in Australia. This goal reflects recognition internationally that chronic diseases are a serious and urgent population health problem [31] which, despite their complex aetiology, are largely preventable through efforts to influence lifestyle-related behaviours such as smoking, alcohol use, nutrition and physical activity [32].

The Prevention Centre is a large partnership of researchers, policy-makers and practitioners. At the outset, it included 31 Chief Investigators, including 17 from academic research environments, 11 from practice and policy environments, and 3 working across both. At the time of writing, the collaboration has expanded to include over 150 individuals who are implementing 40 separate but interconnected research projects. The Prevention Centre had resources (dollars and in-kind) of A$22.6 million over 5 years provided by the NHMRC, Australia’s federal department of health, two state/territory departments of health and a national private health insurer.

As outlined in Table 1, effective governance, supportive architecture, appropriate resourcing and influential leadership are recognised as crucial to successful KMb endeavours [25, 27, 33, 34]. Reflecting this, governance and leadership structures within and across the Prevention Centre are founded on three core elements, namely (1) a Governance Authority, comprising representatives from all funding partners who meet at least quarterly to review and approve research priorities and budgets; (2) a Leadership Executive, comprising policy-makers and academics who provide leadership and stewardship of the overall funding and performance of the centre; and (3) a Scientific Advisory Committee with international membership who function as an external reference group to advise on overall scientific direction.

Operationally, the Prevention Centre functions across three groups where individuals may have multiple and changing roles. A Coordinating Centre manages the Centre business, including project initiation, funding and accountability, and delivers strategies to enable the partnership, including capacity-building, communications and engagement, integration of projects and learning, and Centre evaluation and improvement. Four Standing Capacities – small hubs of individuals with specific expertise in complex programme evaluation, communications, systems science, and implementation and evidence synthesis – provide input across the Centre and lead a number of research projects. Finally, the research is conceived of and implemented by interdisciplinary research project teams.

The Prevention Centre’s initial work plan was founded on three broad ‘planks’, namely (1) partnership research as a vehicle for KMb, with a focus on co-production between academics, policy-makers and practitioners; (2) building knowledge and skills through formal and informal knowledge exchange and capacity-building activities that encourage reflective practice and creative responses to system improvement; and (3) actively designing and cultivating the use of research to impact at a systems level. To operationalise this approach, a range of strategies were used to foster connectivity across stakeholders, encourage co-production of research and build the capacity of members, with the ultimate goal of producing new knowledge and methods, and implementable policy-relevant advice that would influence decision-making in chronic disease prevention.

### Aims

In this paper, we seek to contribute to the growing discourse on partnership approaches to KMb by describing the operationalisation of the Prevention Centre [29, 34]. We use key stakeholder interviews to explore how the Centre is functioning in practice and to develop a conceptual model of KMb within the Prevention Centre. Our aim is to increase understanding of the realities of operationalising KMb strategies within a research partnership. We present this information together with reflections on some key challenges and strategies for tackling them.

## Methods

### Methodology

This study was undertaken as part of the Prevention Centre’s ongoing developmental evaluation [35]. Here, our goal was to identify constructs that appeared to be essential to the operations of the Centre, and to develop a model that reflects the constructs and their relationship with each other. The purpose was to (1) highlight critical areas, including potential leverage points, so that those involved in running the Centre could focus their attention pragmatically for maximum benefit, and (2) use the model to design more rigorous evaluation strategies going forward. Consistent with systems thinking, we strove to investigate the messy realities of how the Centre was operating in practice (emergent patterns), rather than attempting to impose a pre-defined framework [36]; thus, we treated the information in Table 1 as sensitising concepts to be tested with stakeholders [37].

Given the relatively exploratory nature of this investigation, and the need for a high-level strategic view of the Centre’s operations over the past few years (to be supplemented later with perspectives from other stakeholders), our chosen research method was in-depth interviews with the Centre’s Chief Investigators at the mid-point of the Centre’s first 5-year funding cycle.

### Interview participants

All 31 Chief Investigators listed on the original funding proposal were invited to participate in semi-structured interviews; 26 (84%) agreed to participate (Table 2).

**Table 2:** Overview of interview participants

Participants	Who this participant group includes	Number of interviewees
Academics	Chief investigators based at universities and research institutes whose primary roles are in academia	17
Funding representatives	Chief investigators based in government and charitable organisations that co-fund the Prevention Centre and who act as formal representatives for these funding partners; most of them are policy-makers	5
Policy and practice partners	Chief investigators who are primarily based within policy and practice settings (e.g. within government health departments)	4

Many of the academics had previously worked (or still had roles) in health service delivery or policy agencies, and some of the funding representatives and policy/practice partners also held adjunct positions in universities. All had management or executive roles in their organisations and, as Chief Investigators, were leading programmes of research and/or contributing to strategic decision-making in the Prevention Centre.

### Data collection

Interviews (*n* = 26) were conducted between January and March 2016. They took place by telephone (*n* = 20) or face-to-face (*n* = 6) depending on stakeholder location and preference. Interviews were audio-recorded and transcribed verbatim. The average length was 42 min (24–76 min).

Open-ended interview questions were based on existing frameworks that had guided the establishment of the Prevention Centre and its approach to evaluation. This established some a priori concepts, including those identified in Table 1, plus dimensions of research contribution identified in the Payback Framework [38], the breadth of research outputs listed in the United Kingdom Research Excellence Framework [39], forms of research capacity and influence described in the Canadian Academy of Health Sciences Framework [40], and applied categories of influence developed for the Research Impact Framework [41]. The interviews explored stakeholders’ expectations of their involvement in the Prevention Centre and their perceptions about what aspects of the partnership were working well or needed improvement, how the partnership developed and how it functions in relation to the concepts above, if/how co-production was occurring in action, and the degree to which the Centre was producing new knowledge, methods and implementable policy-relevant advice, and influencing decision-making. The interviews were conducted conversationally to allow interviewees to raise issues and explore new ideas. Interviewees were asked to critique core hypotheses about what mechanisms were operating, provide concrete examples from their own experience about how the partnership was working (or not), and were encouraged to provide frank feedback. They were prompted to critique the language used (e.g. ‘co-production’ may be perceived quite differently) and to identify key concepts, challenges and areas for improvement.

### Data analysis

A thematic data analysis approach was used in which patterns and themes within and across interviews were identified [42]. As is usual in the process of constant comparison, analysis was performed in parallel with data collection so that emerging ideas could be explored in further interviews [43]. During this phase of data analysis, two researchers (SR and KG) read the transcripts and developed initial codes across the available dataset. These codes were then reviewed and considered in relation to the key concepts underpinning the Prevention Centre’s initial approach to KMb (co-production, partnerships, and capacity and skills) and intended outcomes (new knowledge and methods, policy-relevant advice and influencing decision-making), and amended in response to further data. This combination of inductive and deductive analysis allowed us to explore novel concepts related to the functioning of the Prevention Centre, and to critique and refine the a priori KMb concepts we had identified. Revised codes were applied to the whole dataset and emerging results were also discussed with members of the wider research team (SW, AW, MO and JS), who had operational roles in the Centre.

Draft findings were presented and discussed at a number of Centre events where interviewees were in attendance, and the authorship team (all but one of whom were also members of the partnership) engaged in iterative discussion over several drafts of this paper, all of which helped to refine the results for accuracy and trustworthiness. Table 3 in the appendix describes other aspects of the study design and conduct according to the consolidated criteria for reporting qualitative research (COREQ) checklist [44].

## Results

**Fig. 1:**
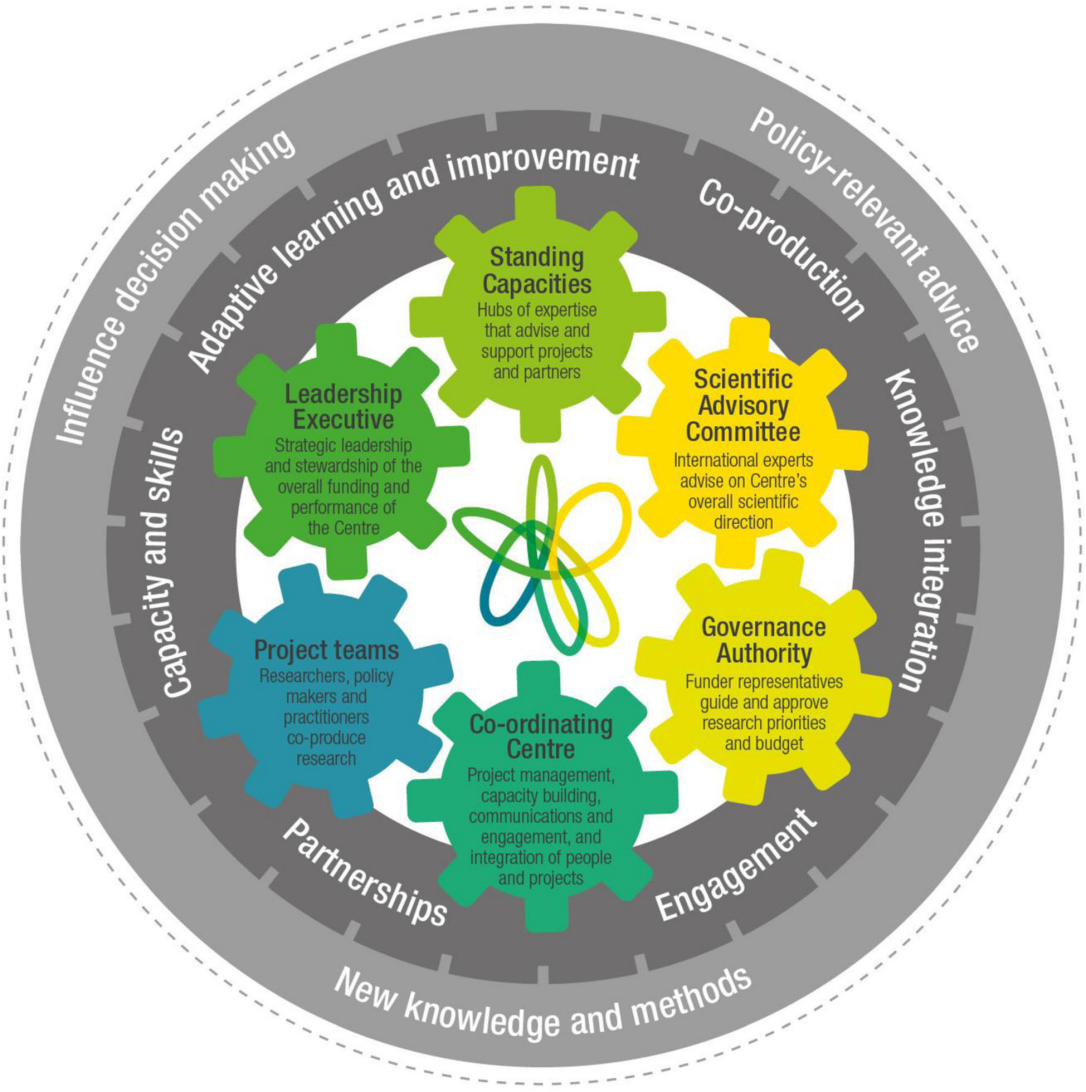
The Australian Prevention Partnership Centre model

We identified six key mechanisms that appear to be crucial to the functioning and potential success of the Prevention Centre as a KMb endeavour, namely Engagement, Partnerships, Co-production, Capacity and Skills, Knowledge Integration, and Adaptive Learning and Improvement (Fig. 1). While the extent to which these mechanisms were being operationalised within the Centre varied, with more work needed in some areas, they were all considered to be essential for reaching our goals. In the following sections we present results in relation to each of these mechanisms, including their role in KMb, and stakeholder reflections on key challenges and achievements to date. We include illustrative comments from stakeholders, labelled to indicate the participant number (e.g. P01) and the interviewee’s connection to the Centre (i.e. Policy or Practice Partner, Funding Representative, or Academic).

### Engagement

The Prevention Centre is made up of a large and diverse network of stakeholders working in a variety of settings (academia, policy, practice and industry) and geographically dispersed across Australia. For many interviewees, the Prevention Centre network was valued as providing a ‘critical mass’ in chronic disease prevention by connecting people who are otherwise separated by institutions, disciplines and geography. Interviewees recognised that this wider group of stakeholders must have a voice in Centre decision-making and scope to actively participate in its work if they are to remain engaged and make valuable contributions.

Engagement was conceptualised as committing time and resources to the work of the Centre, participating in its activities (including undertaking research projects and attending meetings and events), feeling connected with the Centre both in terms of knowing what is happening (e.g. research projects, people, progress) and having a sense of ‘identity’ as a Prevention Centre member, and sharing a common vision for the Centre and its overarching aims.

Interviewees, all of whom were Chief Investigators in the Centre, reported a number of expectations of the Prevention Centre and varied reasons for becoming involved. For some, the appeal of the Centre lay in forging relationships between research and policy, and the opportunity to be part of a national network of people advancing chronic disease prevention. For others, it was an opportunity to try innovative ways of working:

“*I embarked on it as an experimental process to work with the area of prevention … that might generate new information. So that I didn't have an absolute expectation that it was going to go down a particular track … I thought that was a very interesting idea, so that I was very happy to be engaged in the experiment.*” (P01, Academic)

Many interviewees in policy and practice settings saw the Centre as a resource providing tools and expertise, particularly researchers who could give advice and work collaboratively to address policy questions. For some, the value of the Centre also lay in the opportunities it created for thinking and acting outside of the usual constraints of daily responsibilities:

“*I suppose there's a bit more space to think, that sort of blue-sky-thinking …. When you're working with government, for instance on commissioned projects, there's very little opportunity to do that. The questions are very specific and very much set beforehand … I think having a Centre like this allows you to not be constrained by the very immediate outcomes you need to generate through that sort of research and that you can think a bit more about methodology and translational issues*.” (P10, Policy/Practice Partner)

This, in turn, could be motivating and create additional work satisfaction:

“*Being involved in the projects it is very positive from an input perspective. It is very energising as a policy-maker to be involved in those discussions. That brings some quality of working life for me, which is an unexpected benefit*.” (P18; Policy/Practice Partner)

There was considerable variation in the degree to which interviewees felt they had engaged with the Centre, and the extent to which their level of involvement met their expectations. Engagement was viewed as a learning curve in which it took time to get used to the Centre’s work practices and complexity:

“*When the work plan was developed, I wasn't sure or confident I had much to contribute … Then more recently it's much clearer. I have a clearer understanding of what the Centre is about, what the opportunities are for people to contribute, and what I can contribute in the way that I expected initially*.” (P22; Academic)

Those who felt they had a clearly defined role, or had received Centre funding for a specific project, tended to feel more engaged. Conversely, those who saw their involvement as more limited often reported a lack of projects that aligned with their expertise, or uncertainty about how they could be involved. Communications, such as regular centre newsletters, had reportedly enabled many new members in policy and practice settings to find out about projects.

Interviewees argued that compelling strategies to maintain the involvement of “*talented people*” were necessary because of their competing priorities and busy workloads.

“*One of the downsides of having the talented people that we have on board is that they’re supporting other lives. They are supporting their activities, their centres, their businesses, etc. They only have a limited amount of time to commit to the centre. That's been a challenge for us. The directorate has to take a greater role in trying to get those people to contribute more…. The people we have involved are senior and they're talented but because they are* [they have many] *other things they have to do. We are just a small part of that.*” (P21; Academic)

### Partnerships

Cross-sector partnership research was regarded as critical for developing policy- and practice-relevant research. The Prevention Centre was perceived to be doing well in this regard. Interviewees valued the Centre’s approach to building partnerships that spanned personal and professional relationships between individual researchers, policy partners and practitioners, as well as relationships between the Prevention Centre as an entity and other organisations (including universities, government agencies and private companies).

These partnerships were seen to serve a number of functions. They allowed policy-makers to have a voice, influence the research process, and gain access to expertise and resources; they improved communication of research and resources; they facilitated sharing of ideas and collaboration amongst individuals who might not otherwise have been connected; and they generated synergistic dialogue:

“*I saw* [the Prevention Centre] *as an opportunity … to demonstrate a different way for researchers and practitioners, and particularly government, to work together. In particular that this might lead to … better informed research in terms of that research better informing decision-making …. When we get people in the same room, there's definitely a sense of people firing off each other that you get that whole range of different ideas in the room*.” (P21; Academic)

Existing relationships that pre-dated the Prevention Centre were considered crucial in establishing the Centre’s networks. Face-to-face events helped strengthen these relationships and forge new ones, especially when they allowed for informal discussion and connections, but some had difficulties attending such events due to lengthy travel times and other commitments.

While interviewees felt there had been opportunities for researchers, practitioners and policy-makers to interact and learn more about each other’s worlds, many of the non-academics commented on the need to improve cross-sector understanding, identify the value-proposition of projects, and better engage policy-makers in project discussions:

“*There was an assumption from the academics that because it was research it must be good, whereas policy people’s time is very short and they need to understand what's in it for them to actually engage. Again, I think both of us made assumptions…. We've both got to understand each other better to make that sort of co-investigation work go more smoothly*.” (P05; Funding Representative)

A small number of respondents suggested that a stronger, more sustainable network could be built by engaging a wider range of people and sectors, including primary healthcare, government departments such as planning, transport, and sport and recreation, jurisdictions that did not provide funding for the Centre, and other researchers working in prevention. They argued that the Centre offered a rare opportunity to build partnerships across programmes of research rather than ad hoc projects:

“*If we're going to be effective about bringing about change, we have to build relationships between the researchers and policy-makers that are long term … As long as we keep doing it on a one-off project basis, then we don't really have the opportunity to learn and unpack things in the complicated way that many of the issues that we are dealing with deserve. I was interested in seeing whether the Prevention Centre, because it's got longer-term funding, it isn't just project-based, could create a new model where we are able to maximise that long-term partnership*.” (P23; Academic)

### Co-production

Co-production, whereby researchers and decision-makers collaborate in all stages of the research process [45] was recognised as a key driver for generating relevant knowledge and facilitating its use in policy and practice [25, 46, 47]. All interviewees who were active in Centre research identified examples of co-production within their own projects, although there was variation in how this was being undertaken. Examples of co-production included research ideas coming directly from policy-makers, joint development of research questions by policy-makers and researchers, and consultation with policy-makers to inform the development of research projects followed by periodic meetings to discuss the research process and findings. Importantly, some key decisions were being made collaboratively:

“*The idea for the work that we're doing came from a policy-maker and…. the people that we got involved, those policy-makers, all have an interest in this. We said, this is what we've got in mind to do; what're your thoughts on it?... we had an initial workshop with the policy-makers and agreed on what we would do*.” (P13; Academic)

However, policy interviewees tended to report less uptake of policy-driven projects than expected, and poorer collaboration in critical decision-making:

“*I think it's been very mixed. I think some we've helped develop the research questions but mostly the researchers are the ones who come forward with the questions. We've then had drafts to comment on but by the time you get draft as a proposal you've already ... it's a bit late. You haven't sat and brainstormed the research questions first together*.” (P05; Funding Representative)

Facilitators of co-production in the Centre described by interviewees included access to a network of collaboratively orientated academics and policy-makers working in prevention, and formal requirements such as the need to specify policy/practice partnerships in research proposals and an approvals process in which research proposals were vetted by funding partners.

Key challenges in co-production were its time demands, for some representing a significant change in how they undertake research. For example, some academics said they had fewer publications due to investing time in partnership-building and delays in their work because of shaping timelines around dialogue with policy-makers and practitioners. While most accepted this as integral to co-production, it was a challenge for those working in university systems, particularly early career researchers who were trying to establish their academic track record:

“*These are very senior policy-makers, some of the ones I am working with, you literally can't get them at two weeks’ notice or one week’s notice or three weeks’ notice. You have to get into their diaries a month in advance, so getting all of the right people into the right room to have a meeting that reflects the equity of decision-making that you want takes time. During that time, there are other things that you could be doing. I can understand how it is that researchers end up doing most of the work in partnership research, they end up being the researcher, when in fact it is meant to be a little bit more equitable in the sharing of that role*.” (P16; Academic)

### Capacity and skills

The Prevention Centre strives to build the capacity of individuals, groups and organisations to produce and use prevention research via targeted capacity-building activities and through engaging in collaborative research that facilities mutual learning across individuals and contexts.

Most interviewees saw the Centre’s investment in early career researchers (at both the PhD and postdoctoral level) as a key strategy in developing the next generation of prevention researchers, underpinned by the opportunities provided for these researchers to take the lead on innovative projects. Similarly, involving early career practitioners and policy-makers in the work of the Centre was also seen as valuable in building capacity within the prevention workforce:

“The other thing I really like is the focus on mentoring and supporting young practitioners and researchers so that there's much more sustainable approach to improve policy and practice likely into the future.” (P19; Funding Representative)

Activities focused on systems thinking were seen as beneficial for increasing understanding and capacity, although a need for more resources and tools for systems practice was highlighted:

“*I think they could do more* [activities around systems thinking]. *It's filtered through slowly to me over the first two and half years. I think it's really clear when you go to a national meeting that that's the framework out of which we're operating, and that's what we're trying to achieve. Tuning people in who are not in the core leadership group or connecting everybody into the literature and key resources, that we can look at in our own time, and utilise for other projects, and diffuse into the way in which we work, I think could be better done.… Links to web pages, key theoretical papers, highlighting key theoretical concepts. A must-read list or must-have access to a range of resources ...* [are] *the sorts of things that the Centre could have at our fingertips*.” (P26; Academic)

### Knowledge integration

The Prevention Centre’s programme of work is large and ambitious, with 40 projects spanning a range of areas relevant to chronic disease prevention, including urban planning, food systems, alcohol and public health law, as well as implementation, scale up and evaluation.

In reflecting on the Prevention Centre’s goals and progress, many interviewees were concerned that the Centre needs to achieve more than the isolated outputs of separate projects. Rather, it must work towards developing a comprehensive understanding of the different facets of, and possible solutions for, chronic disease prevention. Interviewees noted that interactive forums were helping to connect people across projects:

*“I think some of those ‘sharing forums’ have really brought together lots of different lessons learned and helped discuss those and bring those in the open … where there has been a lot of chance for everyone to put their lessons learned forward or their views forward and share and come up with an agreed way forward together. I think that type of facilitation has been useful.”* (P05; Funding Representative)

But they also argued that the Centre needs to do more to consolidate its findings across projects:

“*I find in terms of governance what we have is an executive committee that makes decisions, and then we have project leads, but what I'm not seeing really is the middle management governance or what I would call stream leaders. If there's a coherent stream of work, who's leading that? ... I know there are people that have overview of areas of work, but it doesn’t present as a coherent stream of work that connects and answers big questions and is actively driven by a stream leader. It's a bit more laisse faire and it's much more focused around individual projects which come together as a loose coalition*.” (P25; Academic)

“*The thing that bothers me more is that I think it might look very scattered. We gave some money to this person and they did this and that was good and it got published. Somebody else did something else over here. I'm worried that it's going to lack some kind of central organising thing that lets people understand. To make the centre more than the sum of its parts*.” (P23; Academic)

Two strategies were suggested for combatting the problem of siloed projects. First, a steering group with responsibility for working across the Centre to identify how the outputs of each project could contribute to a larger story in prevention, and convening theme groupings of researchers working in related areas. Second, to achieve this ‘bigger picture’ there needed to be a clearer overarching purpose towards which everyone in the Centre was working.

### Adaptive learning and improvement

Given the complex, organic and dynamic nature of the Centre, which consists of many actors and components engaged in multiple projects and activities embedded within and across existing research, policy and practice systems, adaptive learning and improvement is crucial for enabling the Centre to develop productively. This involves cycles of reflection on the practice of partnership, feedback and evaluation, and responsive change.

Many interviewees regarded adaptive learning and improvement as vital to the successful functioning of the Centre, and valued efforts to facilitate it. To some extent, the interviews occurred too early in the Centre’s lifespan for respondents to comment on the success of these efforts, but there were some examples of adaption in practice. For example, a researcher commented that the initial work plan seemed rigid, with greater administrative accountability than a traditional project grant, but this had become more elastic over time, allowing for the development of an innovative and responsive programme of work:

“*There's also a sense of if we want to do something really creative we have to actually allow for proposals to come in that aren't part of the plan … I only observe it from the outside but I know that there have been projects that have been submitted, approved and signed off, which I gather allows for much more flexibility than a fixed pre-determined work plan. My experience of it has been there has been more flexibility as time has gone on*.” (P01; Academic)

Another observed that project management within the centre had evolved to better engage academics, in particular by taking a more hands-off approach that enabled them to develop and lead projects.

“*I think we've nudged in the last year towards giving people bits of more specific things to do and letting them get on with it … it's working better in a couple of ways. I think the investigators around the broader Centre are feeling more engaged because they're being given things to do that they can do and then not being scrutinised within an inch of their lives for everything that they do … What we've done I think in the centre is try and orient the areas in which they're doing things to the areas that are around the prevention system that we're trying to research. Then within that, we've just been asking people to get on with things. That's been a better development and some have taken to that quite well*.” (P08; Academic)

### Progress towards Prevention Centre goals

The goal of the Prevention Centre is to produce new knowledge and methods, and implementable policy-relevant advice, that influence decision-making in chronic disease prevention. On the whole, interviewees felt the Centre’s research outputs would be useful and that its model of co-production and research–policy partnerships were valuable in ensuring its work would influence decision-making. However, most considered it to be too early for the Centre to have had any identifiable impact on decision-making:

“*… certainly within our own projects we're not at the stage yet of influencing actual policy decisions. We've got policy people engaged in the work right from the beginning, the plan is that it will influence their decisions. They are keen for the work to influence their decisions. It's not like we're going to knocking on their doors saying, here's our work do you want to take it into account? They're waiting for it with baited breath.*” (P01; Academic)

“*I haven't seen many outputs yet, because we're sort of in that development phase. Certainly some of the pieces of work look like they're going to be highly relevant … Direct applicability, I think I probably need to just wait and see. Most of the work is going to be relevant and useful*.” (P09; Funding Representative)

### Refining our model of KMb

We used the data outlined above to critique and broaden the three broad ‘planks’ that had underpinned the Centre’s initial work plan. The result was a refined conceptual model of how the Prevention Centre operates as a KMb endeavour. This model, illustrated in Fig. 1, maintains the key elements of the original ‘three planks’ but teases apart the concepts to reveal a more delineated model. It distinguishes between (1) the ‘inputs’ of the Centre (the six interconnected cogs in the middle of the figure), which are the governance and organisational structures that underpin the Centre and enact its strategies (as described earlier); (2) the ‘KMb mechanisms’, described above, that drive the Centre’s productivity (the inner circle); and (3) the Centre’s ‘intermediate impacts’ (the outer circle of the model), which is how we expect knowledge to be used as a result of the inputs and proximal impacts. The more distal impacts, for example, increased health promoting behaviours, socioeconomic benefits and improved health outcomes, are intentionally omitted in the model consistent with the understanding that the outcomes of research may take some time to manifest [48]. The model reflects the dynamic nature of our work and the interaction between elements of the partnership such that effects feed both inwards and outwards. The dashed circle encapsulating the framework represents a permeable boundary, recognising the Centre interacts with, influences and is influenced by many factors, including political, policy, health service and university systems.

## Discussion

This paper explores how The Australian Prevention Partnership Centre, a research partnership for chronic disease prevention, has operationalised its approach to KMb. Through interviews with 26 of the Centre’s Chief Investigators, we identified six mechanisms that appear to facilitate KMb within our Centre, namely Engagement, Partnerships, Co-production, Capacity and Skills, Knowledge Integration, and Adaptive Learning and Improvement. Interviewees were supportive of the KMb strategies being enacted and believe they are helping the Centre progress towards its goals. A number of challenges were identified, particularly in terms of researchers, policy-makers and practitioners understanding each other’s contexts, maximising policy contributions, and ensuring that knowledge gained from projects and players across the Centre is integrated to provide a ‘bigger picture’ in chronic disease prevention. At the time of undertaking the interviews, it was too early to identify how the Centre was performing in terms of its desired impacts, but early indications were positive, with many noting that the Centre was on its way to influencing policy and practice decision-making.

Through interviews with key stakeholders at the mid-point of the 5-year funding cycle, and drawing on our engagement with the literature, we critiqued and broadened our early conceptual model to develop a refined understanding of how the Prevention Centre operates as a KMb endeavour. This model recognises that the creation and use of research for complex problems like chronic disease is optimised when diverse members share goals and contribute to and identify as part of the partnership (Engagement) [23, 33, 49]; strong networks are built across disciplines, organisations and geography (Partnerships) [15, 24, 33]; opportunities for researchers, policy-makers and practitioners to work collaboratively and share decision-making are created and supported (Co-production) [25, 46, 47]; and there is capability development for members to engage with cross-sector partners and to work in new and creative ways (Capacity and skills) [27, 28]. The KMb model also highlights the need for processes that draw together different kinds of knowledge generated across the Centre so that it achieves more than the sum of its parts (Knowledge integration) [50]. Finally, our model highlights the value of engaging in continuous reflection and adaptive learning to ensure the Centre’s operations and research outputs are fit-for-purpose in this complex and dynamic environment (Adaptive leaning and improvement) [23, 51, 52]. The KMb mechanisms identified within our conceptual model are supported by the organisational and governance structures of the Prevention Centre.

### Strengthening our approach

In addition to refining our conceptual model of KMb, the findings from these key informant interviews have provided opportunities for reflection and action to strengthen strategies for facilitating KMb. For example, on the basis of the broader findings, the Prevention Centre has taken steps to better tailor communication of evidence to the needs of policy-makers, including the development of concise and accessible ‘Findings Briefs’ that summarise key findings from completed projects. To strengthen knowledge integration, we have also established a number of theme groups that bring together project teams whose work falls within a similar area to discuss possible synergies and identify how their individual project findings can contribute to a more integrated ‘bigger picture’.

### Using the model to inform ongoing evaluation

Articulating our model of KMb was challenging due to the multifaceted and dynamic nature of KMb and the complexity of the Prevention Centre itself, but it was worthwhile, particularly because the model is now guiding further developmental evaluation strategies [35]. For example, constructs in the model underpin our regular partnership surveys and have been used in evaluation interviews with researchers, policy-makers and practitioners. These interviews are also being used to ‘test’ the constructs and their relationship to each other, enabling us to refine the model so that it better reflects stakeholders’ experience of practice realities [53]. We note, however, that partnerships evolve over time and that different mechanisms and strategies may be more important in different phases [33].

### Implications for other KMb partnerships

Our model was developed to describe the Australian Prevention Partnership Centre’s operationality. As such, it reflects the Centre’s particular goals and context, including partners, organisational needs, funding requirements, and state and national infrastructures. Nevertheless, we suspect that it is broadly applicable to other partnerships, partly because our mechanisms align with other descriptions of success factors in KMb and collaboration. For example, a recent report, which combined two reviews of reviews examining the effectiveness of interventions to increase the use of research in decision-making, identified six mechanisms through which research-informed policy and practice might be achieved. These were (1) building awareness for, and positive attitudes towards using research; (2) building agreement on policy-relevant questions and fit-for-purpose research; (3) communicating and providing access to research; (4) facilitating interactions between decision-makers and researchers; (5) developing skills to access and make sense of research; and (6) influencing decision-making structures and processes [54]. These are largely consistent with our findings. The ‘cogs’ that depict the Centre’s governance and organisational structures in our model are probably specific to our project, but some forms of governance and day-to-day management will likely be essential in any partnership [22, 55, 56].

## Conclusion

The Australian Prevention Partnership Centre is a large, national research–policy–practice collaboration with a remit to develop information, tools and actions needed for effective systems-level prevention of lifestyle-related chronic disease. Work undertaken as part of our evaluation enabled us to articulate our approach to KMb in the form of a conceptual model which is now being used to inform a mixed-methods evaluation framework that is exploring not only what we have achieved, but how, i.e. the processes that contribute to (or hinder) the advancement of our goals. This evaluation forms an integral part of our engagement in adaptive learning and improvement and, we hope, will also contribute to the value, functionality and operationalisation of other research partnerships.

## Appendix

**Table 3:** Additional information about the research methods as per the consolidated criteria for reporting qualitative research (COREQ) checklist

Personal Characteristics 1. Interviewer/facilitator, 2. Credential, 3. Occupation, 4. Gender, 5. Experience and training	Interviews were conducted by author SJR, a researcher with 10 years of qualitative research experience, including the conduct of in-depth interview studies for her PhD and subsequent work. SJR was a newly employed research fellow with the Prevention Centre at the time of the interviews, supervised by SW
Relationship with participants 6. Relationship established, 7. Participant knowledge of interviewer, 8. Interviewer characteristics	SJR knew a few of the interviewees professionally. Participants all knew SJR was employed by the Prevention Centre and was leading the Centre’s evaluation. To reduce social desirability bias, interviewees were reassured that transcripts would only be read by members of the immediate research team, and that the full author team would only view de-identified quotes
Theoretical framework 9. Methodological orientation and Theory	The study was part of a developmental evaluation [35], underpinned by systems thinking [27, 57] and realist ontology [58]. The concepts that informed the interview questions and framed the evaluation approach were informed by the studies of collaborative research partnerships outlined in Table 1
Participant selection 10. Sampling, 11. Method of approach, 12. Sample size, 13. Non-participation	Because the study focused on perspectives of the Centre’s Chief Investigators, purposive sampling was used. Participants were invited by email with one reminder. There were 31 possible participants, 26 of whom (84%) agreed to take part. Four potential participants declined to participate due to poor availability, and one did not reply to the invitations
Setting 14. Setting of data collection, 15. Presence of non-participants, 16. Description of sample	Interviews were conducted between January and March 2016. Interviews took place either face-to-face in the interviewee’s place of work (*n* = 6) or by telephone (*n* = 20), depending on their location and preference. The sample of interviewees is described in the methods section
Data collection 17. Interview guide, 18. Repeat interviews, 19. Audio/visual recording, 20. Field notes, 21. Duration, 22. Data saturation, 23. Transcripts	There were no repeat interviews. Field notes were not taken because interviews were audio-recorded and professionally transcribed. The average interview length was 42 min (range, 24 to 76 min). We did not take a data saturation approach, and therefore all available interviewees within our sample were interviewed to obtain the widest range of possible views and experiences. See methods section for further information
Data analysis 24. Number of data coders, 25. Description of the coding tree, 26. Derivation of themes, 27. Software, 28. Participant checking	Data was coded by two researchers using inductive and deductive strategies, the latter identified from our literature search. Data were coded by SR in NVivo 10 qualitative data management software [59], and SR and KG discussed emerging themes on an ongoing basis with other members of the research team to check for reliability and trustworthiness of analysis. Other details are described in the methods section
Reporting 29. Quotations presented, 30. Data and findings consistent, 31. Clarity of major themes, 32. Clarity of minor themes	We report on the results using illustrative quotes. Only major themes (i.e. those that serve the purposes of this this study) are presented here. Consistency between the data and findings presented was checked as part of the iterative cycles of constant comparison in our analysis
